# The Poggendorff illusion in Ruben's Descent from the Cross in Antwerp: Does the illusion even matter?

**DOI:** 10.1177/20416695221125879

**Published:** 2022-10-11

**Authors:** Olga Daneyko, Natale Stucchi, Daniele Zavagno

**Affiliations:** Department of Psychology, Sociology and Politics, 7314 Sheffield Hallam University, Sheffield, UK of Great Britain and Northern Ireland; Department of Psychology, 9305 University of Milan-Bicocca, Milano, Lombardia, Italy; NeuroMI, 582600 Milan Center for Neuroscience, Milano, Lombardia, Italy; Department of Psychology, 9305 University of Milan-Bicocca, Milano, Lombardia, Italy; NeuroMI, 582600 Milan Center for Neuroscience, Milano, Lombardia, Italy; BiPac, Centro Interdipartimentale di Ricerca sul Patrimonio Artistico e Culturale, Milano, Lombardia, Italy

**Keywords:** Poggendorff illusion, Rubens, Descent from the cross

## Abstract

Two experiments are described, the purpose of which was to investigate the presence of a misalignment illusion caused by Poggendorff-like conditions in two paintings by Peter Paul Rubens, both depicting the *Descent from the Cross*, one located in Antwerp (Belgium), the other in Lille (France). The first shows a geometrical misalignment made by Rubens in a minor detail, which is considered proof that the artist observed the Poggendorff illusion. The second painting, instead, shows a perfect geometrical alignment in a similar detail. In experiment 1, participants were asked to align a top segment to a lower one in two types of stimuli: a full-size digitally manipulated reproduction of the painting and a Poggendorff-like configuration that recalled the painting's lines displacement and tilt. Adjustments were performed from two distances, one up close (painting distance) and one from below and far (observation distance). Results confirmed the presence of the Poggendorff illusion, but mean adjustments significantly differed from the misalignment perpetrated by Rubens. Experiment 2 was set up in a similar fashion with the Lille painting. Results confirmed the presence of the Poggendorff illusion also in this painting; however, the alignment by Rubens coincides with the geometrical one. Results from both experiments do not support the claim that Rubens observed the Poggendorff illusion and therefore corrected for it in the Antwerp painting. An alternative account is discussed, which relates to the structural layout of the painting.

## Introduction

**Figure 1.**
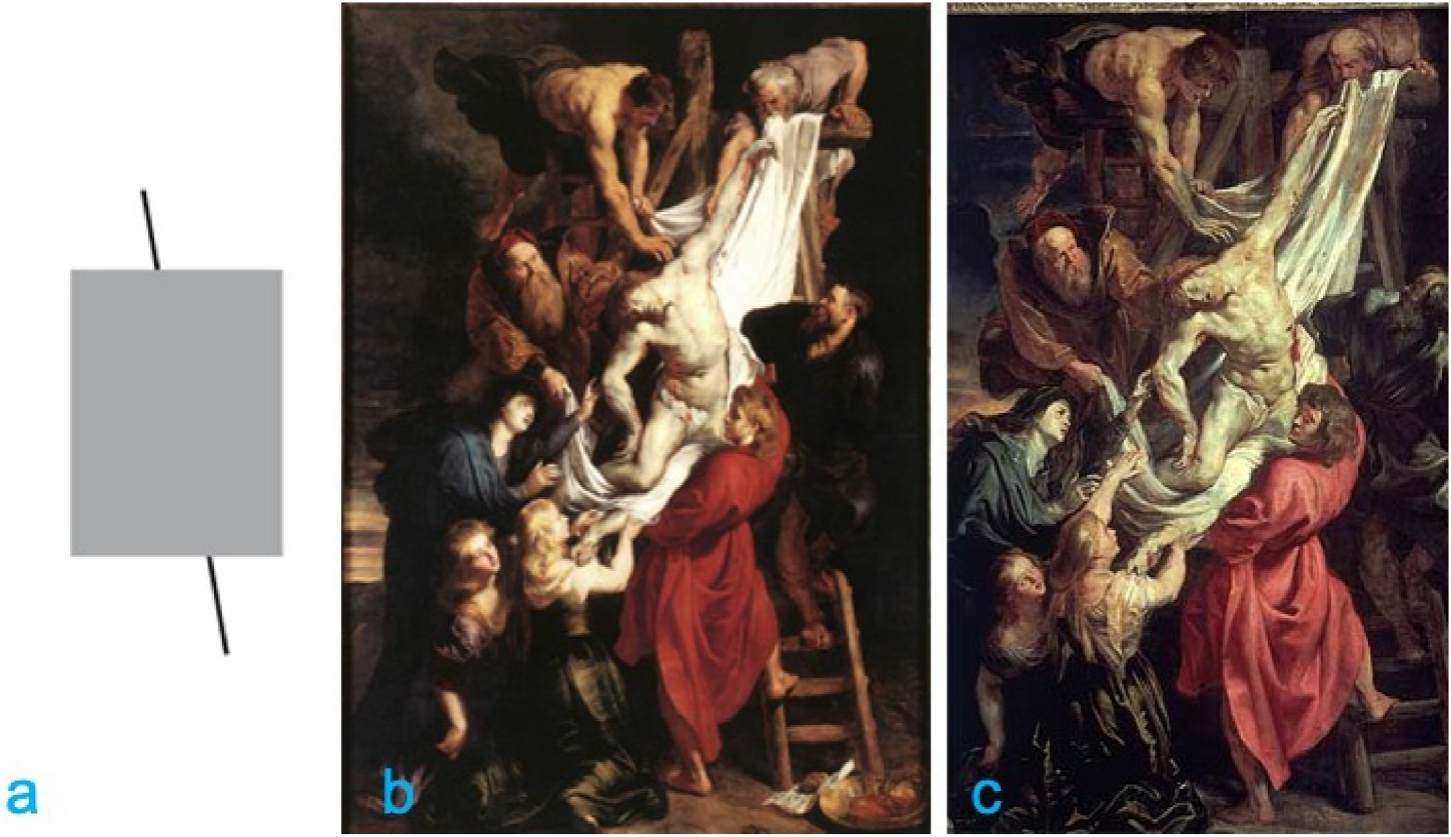
(a) A Poggendorff configuration derived from Ruben’s *Descent from the Cross in Antwerp* (b). (c) Ruben’s preparatory study is now conserved in the Courtauld Gallery (The Courtauld, London, Samuel Courtauld Trust).

While admiring artwork, it is not uncommon to wonder why an artist adopted certain solutions instead of others. In some cases, partial answers can be found in documents left by the artist, such as notes, sketches, preparation studies (e.g., Arnheim, 1962), or even specific art theory treatises (e.g., Da Vinci, 1804; Kandinsky, 1977; Lomazzo, 1584; see Zavagno et al., 2022). Sometimes, however, there are questions that arise from observing a specific artwork, the answer to which cannot be found in written documents. Here we address the issue of whether Peter Paul Rubens (1577–1640) was aware of the Poggendorff illusion (Figure 1a) while painting his famous masterpiece *Descent from the Cross* (Figure 1b) for the Cathedral of Our Lady in Antwerp. In this painting, the two visible portions of the ladder's right-side rail, partially occluded by one of the characters, are geometrically misaligned. In 1984, by comparing the painting with its preparatory study, the Courtauld oil sketch (Figure 1c) in which the two visible portions of the ladder's right-side rail are instead geometrically collinear, Topper concluded that Rubens discovered the Poggendorff illusion. Ever since, Topper's (1984) conclusion has been taken for granted as if it were ground truth (e.g., Ninio, 2001; Solso, 2003).

Following Topper's hypothesis, the geometric misalignment in the Antwerp painting should be considered as an attempt to achieve a perceptual alignment to overcome the Poggendorff illusion. The hypothesis is indeed fascinating and rather convincing, as there are not many paintings for which a perceptual alignment has been preferred over a geometrical one (Zavagno et al., 2015). Actually, the only other case we are aware of can be observed in the *Lunetta di San Lorenzo* (before 450 AD, Mausoleum of Galla Placidia, Ravenna), in which the martyrdom of Saint Lawrence is represented. The saint is depicted crowned in a golden halo, holding a book in his left hand and a long golden rod cross in his right hand. The cross leaning on his shoulder slips behind his neck from which it becomes visible again as it intersects the saint's halo. The intersection was rendered by the mosaicists with a transparency effect, a truly fascinating artifice created to show the integrity of the cross (Zavagno, 1996). Moreover, the two visible segments of the cross carried by the saint are not geometrically collinear (Daneyko et al., 2011). By employing an adjustment method, Zavagno et al. (2015) showed that the physical misalignment in the mosaic is consistent with the mean perceptual alignment by participants when adjustments were performed from a position that is consistent with the observation distance in the Mausoleum. Such findings support the hypothesis that the geometrical misalignment of the two visible portions of the long cross was perpetrated to adjust for the perceptual misalignment caused by the Poggendorff illusion.

In their paper, Zavagno et al. (2015) also considered Ruben's case with the Antwerp masterpiece. In their experiment, a thick line was superimposed on the lower visible portion of the ladder's rail, while the upper portion of the rail was digitally removed from the scene and replaced with another thick line. Participant's task was to align the upper line with the lower line, which was fixed. The mean geometrical misalignment by the participants, indeed caused by the Poggendorff illusion, was significantly inferior to Ruben's misalignment. Such findings does not support Topper's claim. However, the experiment was conducted on a computer screen, hence with a gigantic difference in size with respect to the original painting, factor which may account for the discrepancy between the mean adjustments and Ruben's own misalignment. It is known, in fact, that the magnitude of the Poggendorff illusion can be affected by many factors (Vicario, 2008, 2011): the amplitude of the acute angles formed by the abutting oblique lines and the occluder (Gallace et al., 2012); the orientation of the illusion's configuration (Gallace et al., 2012; Green & Hoyle, 1964; Leibowitz & Toffey, 1966; Morgan, 1999); the presence of 3D information (Koning & van Lier, 2007). It is therefore possible that the size of the configuration employed, along with the viewing position of the observer, may also influence the outcome of the illusion.

To test the effect of such factors on the adjustment performance for the illusion in the Antwerp masterpiece, we conducted a new experiment in which we employed the same stimuli as in Zavagno et al. (2015) but projected on a screen, such that the projections matched in size the original painting (422 × 311 cm),

## Experiment 1: The Poggendorff Illusion in the Antwerp Masterpiece

### Method

*Participants:* Twenty people (14 females) with an age range between 18 and 31 years old (M = 25.1, *SD* = 4.63), all studying or working at the University of Milano-Bicocca, participated in the experiment. None of the participants were aware of the purpose of the experiment, but obviously many may have been familiar with the painting and some participants may have had some knowledge of the classic Poggendorff illusion. Participants were not informed about authorship or the title of the painting. Written informed consent was obtained from all participants, in compliance with the tenets of the Declaration of Helsinki. The number of participants meets the sample size (19) required to obtain statistical power of 0.8 with an effect size *f* = .25.

*Material and procedure:* We employed a within-participants design. The three within factors were *stimuli* (two levels), *viewing distance* (two levels), and *repetitions* (six levels).

**Figure 2.**
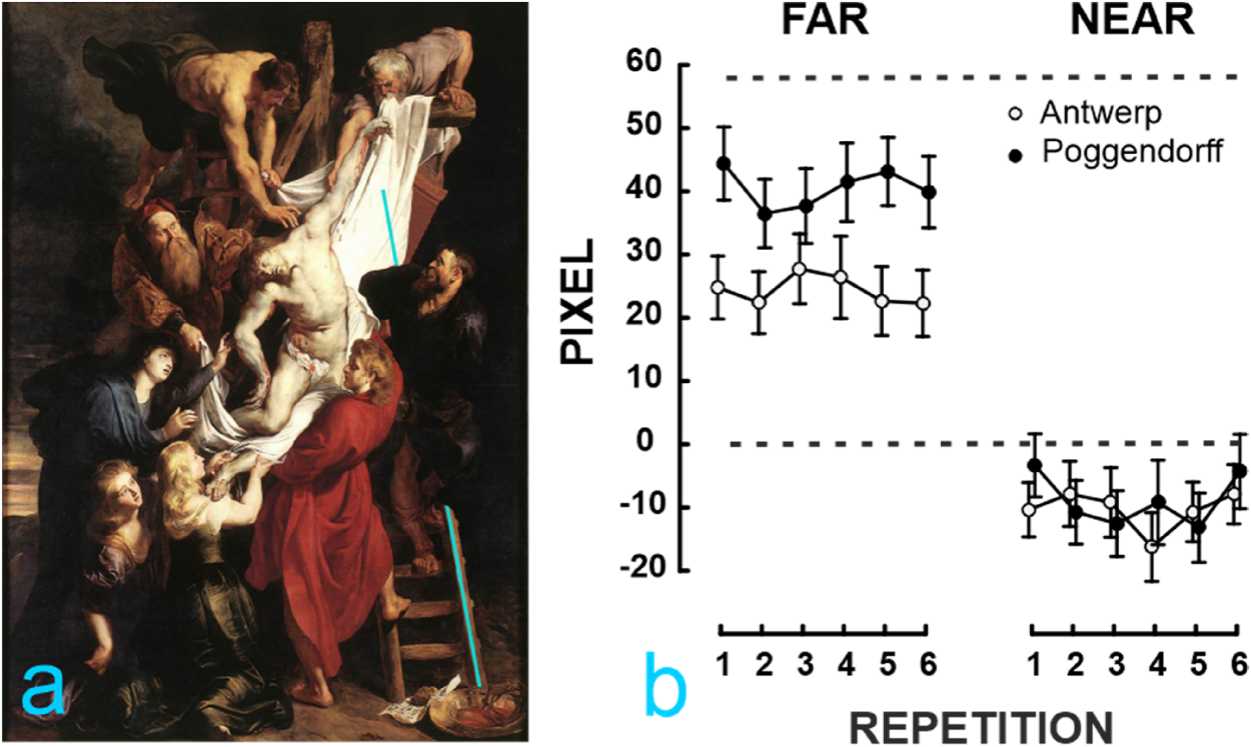
Results for experiment 1. Panel (a) shows the mean adjustment on from 800 cm. Panel (b) shows mean adjustments in pixels for the two types of stimuli (Antwerp and Poggendorff) in the two conditions of observation (far and near). Adjustments of the upper light blue line were repeated six times. Vertical error bars represent standard errors; the top dashed line corresponds to Ruben’s original displacement. Positive values indicate that adjustments were made to the right and negative to the left of the images with respect to geometrical alignment.

The *stimuli* employed were two: (1) a digital copy of the central panel of the Antwerp triptych (412 × 304 cm) similar to the one employed by Zavagno et al. (2015), but projected on a large screen in an auditorium (Figure 1b) and (2) a textbook version of the Poggendorff illusion (Figure 1a) projected on a large screen, designed so that it would match the painting's “Poggendorff” in spatial configuration, inclination, and size. The Antwerp stimulus was digitally modified by removing from the scene the upward portion of the right rail. A thick blue line was superimposed on the bottom portion of the right rail, while the upper portion was replaced by a similar blue line bearing the same inclination. This second line was adjustable in a position sideways.

The factor “*viewing distance”* was set to reproduce two possible viewing positions of the original painting: (1) *near* (∼50–60 cm from the screen), which mimics the position and distance from which the artist would have been painting, and (2) *far* (∼700 cm), which mimics a ground level distance from which the entire painting can be admired in the Cathedral. The order of the two viewing distances was randomized across participants.

With regards to *repetition*, participants performed in random order 12 adaptive adjustments for each stimulus type and distance, 6 starting with the upper line randomly positioned at the left side with respect to geometrical collinearity and 6 starting with the upper line randomly positioned at the right side with respect to geometrical collinearity.

Participants’ task was to achieve perceptual collinearity between the upper and lower blue lines by shifting horizontally the upper one. Participants performed alignments from both viewing conditions using simple vocal commands (left or right).

In the *near* trials, the top blue line was positioned 15 cm above the average height of our observers (168 cm). In the *far* trials, participants performed the adjustments sitting in front of the screen at a distance of 800 cm, with the top blue line 235 cm above the average height of our observers. Trials for the two *stimuli* and *viewing distances* were grouped in four distinct experimental sessions with starting session randomized across participants.

### Results and Discussion

Results for both stimuli are shown in Figure 2a-b. A check for outliers led us to exclude 11 extreme values out of 480 (outlier boundaries were set at ± 2.5 *SDs*). An ANOVA for repeated measures was carried out on the data, with *stimulus*, *viewing distance*, and *replications* as within-subjects factors. Factor *viewing distance* (*F*_(1, 19)_ = 99.95, *p* < .001, η^2^_p_ = .84) and the interaction viewing distance by stimulus (F_(1, 19)_ = 5.30, *p* < .05, η^2^_p_ = .22) determined significant effects. The significant interaction is mainly due to a significant difference between Antwerp and Poggendorff in the far condition, as resulted from a post hoc comparison (Bonferroni, *p* = .01) contrasted to the absence of difference in the near condition, as can be visually appreciated in Figure 2b.

Results confirm that *viewing distance* modulates perceived collinearity of the illusion in the *stimuli* employed. As shown in in Figure 2b, when both *stimuli* were viewed from distance *fa*r, mean adjustments (Antwerp 24.38 px; Poggendorff 40.51 px) are in line with the direction of Rubens’ actual displacement (56 px); when *stimuli* are viewed from distance *near*, mean adjustments (Antwerp M = -10.37; Poggendorff M = −8.89) are shifted to the left with respect to both the actual displacement and the point of the geometrical alignment (0 px). In other words, the perceived alignment from the near distance was contralateral to the adjustments that the Poggendorff illusion would usually require. This may depend on several factors, among which the size of the visual angles with which the painting is observed from up close.

These findings confirm an effect of viewing position/distance in the perceptual outcome of the Poggendorff illusion.

As confirmed by one-sample *t*-tests conducted to compare mean adjustment with the point of geometrical alignment, participants corrected for the Poggendorff illusion in both *stimuli*: Antwerp far t(19) = 4.91, *p* < .001, *d* = 1.09; near t(19) = −2.39 *p* < 0.05, *d* = −.53; Poggendorff far t(19) = 8.04,*p* < 0.001, *d* = 1.79; near t(19) = −1.95, p = .06, *d* = −.43). However, one-sample *t*-tests conducted to compare mean adjustments with the artist's adjustment shows that performed adjustments for both *stimuli* are not comparable to the artist's geometrical misalignment, which was further to the right (56 pxl): Antwerp far t(19) = −6.37, *p* < .001,*d* = −1.42; near t(19) = −15.29 *p* < .001,*d* = −3.41; Poggendorff far t(19) = −3.07, *p* < .01,*d* = −0.68; near t(19) = -14.21, *p* < .001,*d* =−3.17.

**Figure 3.**
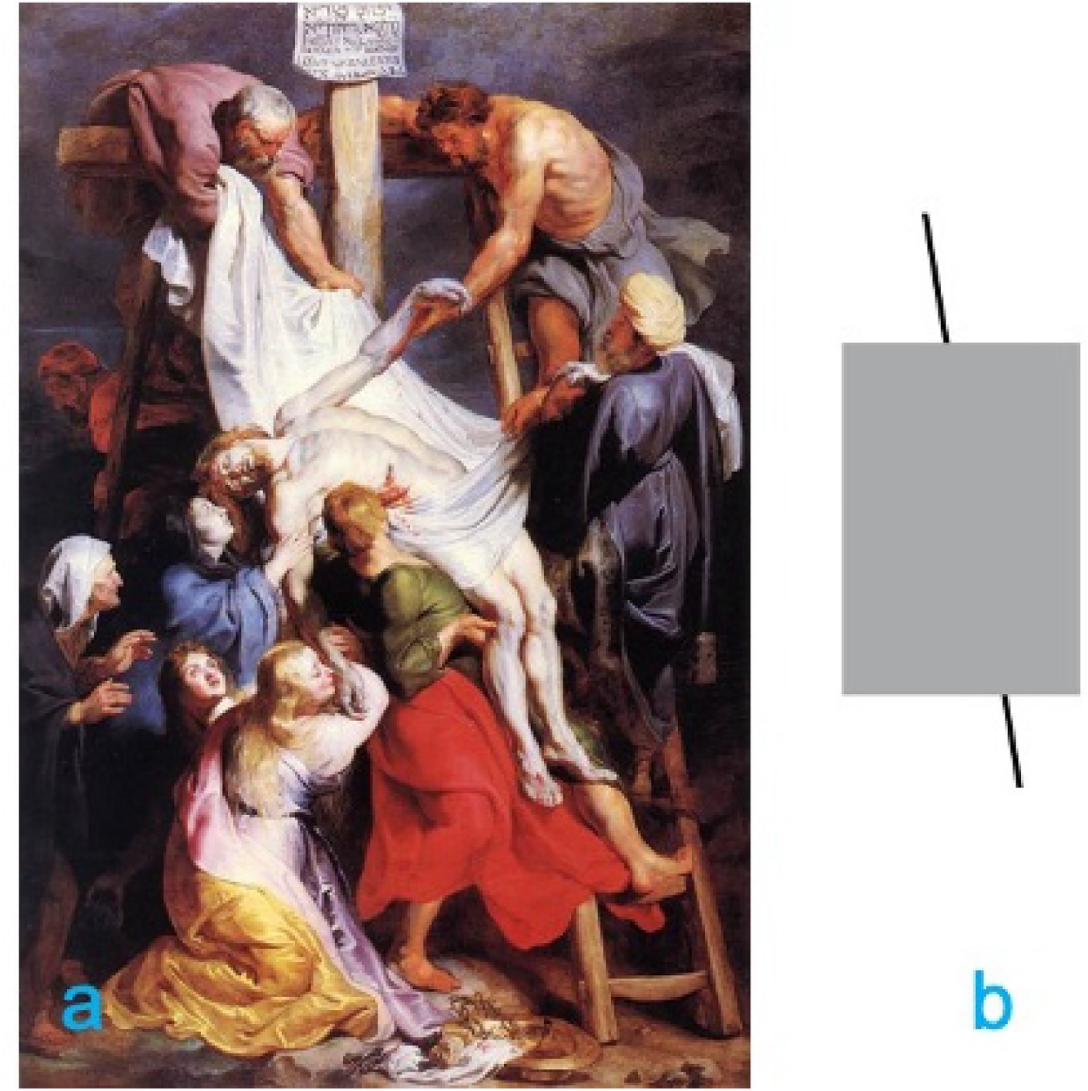
(a) The *Descent from the Cross* conserved in the Palais des Beaux-Arts in Lille, France and (b) the derived Poggendorff configuration.

The fact that mean adjustments are different from the geometrical alignment from both distances confirms the presence of the Poggendorff illusion. Moreover, the position from which alignments were performed deeply affected how the illusion appeared. In particular, adjustments from up close are somewhat closer to the point of geometrical alignment but shifted to the left. Adjustments from afar, instead, are bigger and shifted to the right: the direction is the same as the misalignment by Rubens, but statistically different from it. Such findings do not support the claim that Rubens corrected for the Poggendorff illusion in the Antwerp masterpiece. We, therefore, considered the hypothesis that maybe Rubens perpetrated the misalignment for another purpose. Our hypothesis is that the misalignment was perpetrated to fix an issue related to the displacement of the characters in Rubens’ Baroque composition, which mimics but does not reproduce the exact proportions of the Courtauld oil study. Our hypothesis will be addressed in full in the General discussion and it builds upon a historical fact: just 2–3 years after the completion of the Antwerp masterpiece, Rubens depicted yet another *Descent from the Cross* (now in Palais des Beaux-Arts, Lille) that matches in size the previous painting (Figure 3a). In this other version, the left rail of a ladder is partially occluded but the two visible parts are geometrically aligned. Could it be that the configuration of the Lille painting does not determine a Poggendorff illusion (Figure 3b)? If this second painting induces a perceptual misalignment that however was not corrected for, there are only two possible alternative explanations: either Rubens forgot about misalignment issues altogether, or these were never really an issue to begin with.

## Experiment 2: The Poggendorff Illusion in the *Descent From the Cross* in Lille

If Rubens became aware of the Poggendorff illusion, he probably would have corrected for it also in his works painted after the Antwerp masterpiece, unless, of course, the illusion was not present in those later artworks where the pictorial configuration might yet recall a Poggendorff-like situation. The *Descent from the Cross* in Lille represents therefore an ideal case study, as it was painted in 1616–1617, which is about 2–3 years after the *Descent* in Antwerp was completed. The two paintings are similar in size, but in the *Descent* in Lille, the two visible portions of the ladder's left rail are geometrically aligned.

The two paintings bear important differences in terms of their overall color scheme and their structural layout. Yet, the inclination of the ladders in the two paintings is rather similar, leading to a similar Poggendorff-like configuration. Hence, differences aside, the question is intriguing: does the painting give rise to a Poggendorff illusion?

### Method

*Participants:* Twenty-two people (15 females), with an age range between 18 and 56 years old (M = 26.9, *SD* = 8.10), who did not take part to experiment 1. All participants were studying or working at the University of Milano-Bicocca. Participants completed an informed consent with an overview of the experimental procedure, in compliance with the tenets of the Declaration of Helsinki. None of the participants were aware of the purpose of the experiment. However, some participants may have had knowledge of the classic Poggendorff illusion. The painting, instead, is not much represented in the history of art books and is not easy to find on the web. Moreover, participants were informed about the authorship and title of the painting only after the experimental sessions were completed.

**Figure 4.**
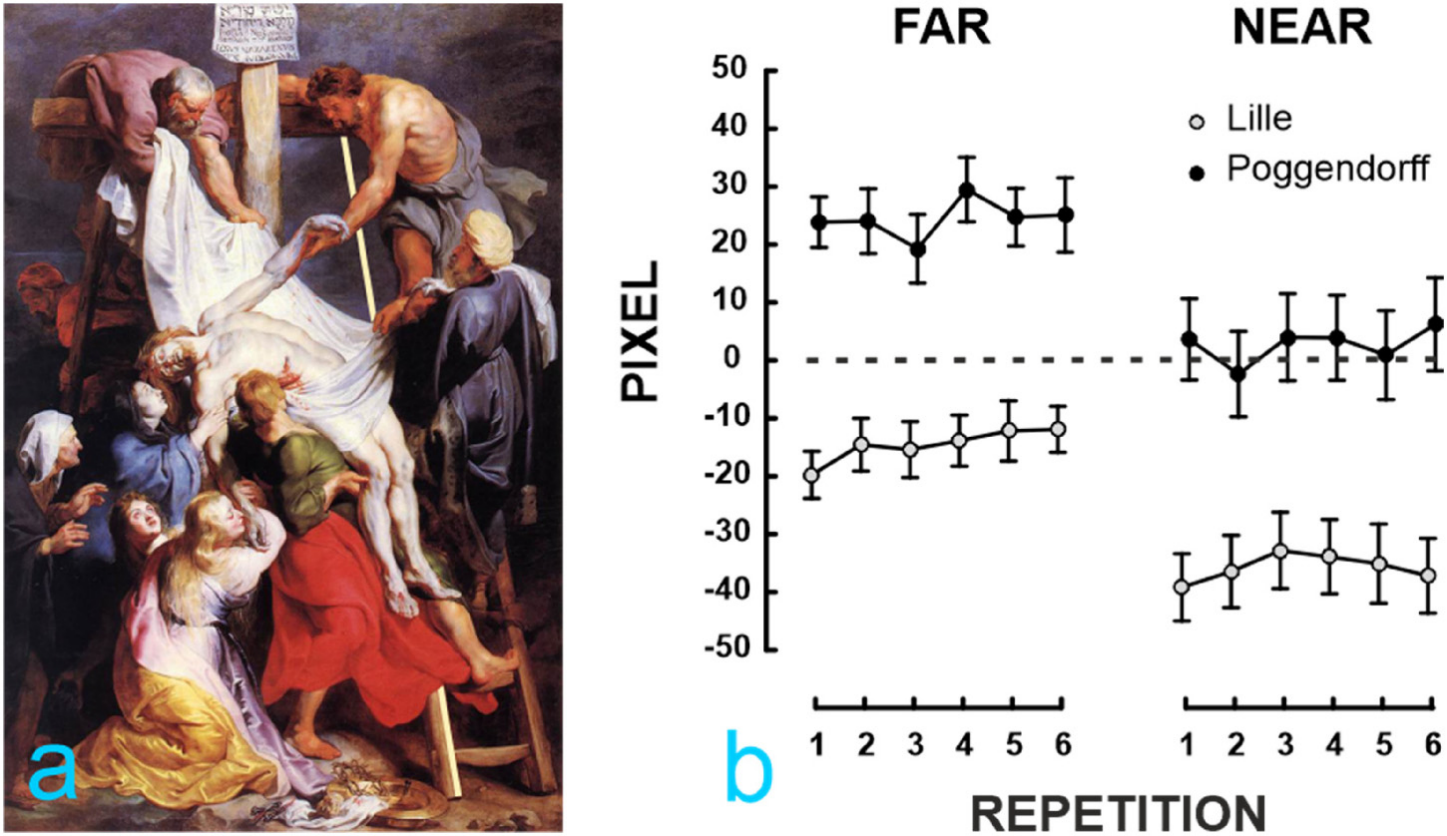
Panel (a) shows the mean adjustment from the distance far. Panel (b) shows the mean adjustments in pixels for the two types of stimuli (Lille and Poggendorff) in the two distance conditions (far and near). Adjustments of the upper light yellow line were repeated six times. Vertical error bars represent standard errors. Positive values indicate that adjustments were made to the right and negative to the left of the images with respect to geometrical alignment.

*Material and procedure:* Apart from the images, the experimental design and its location were the same as in experiment 1, with three within factors: *stimuli, viewing distance*, and *repetitions*. The two sets of stimuli projected on the big screen were a digital copy of the Lille *Descent* and a textbook version of the Poggendorff illusion designed so that it would match the painting's “Poggendorff” in terms of its spatial configuration, inclination, and size (Figure 3b). The *Descent* was digitally manipulated as the Antwerp stimuli in exp. 1 (Figure 4a). The procedure adopted was the same as in experiment 1.

### Results and Discussion

Results for the two stimuli are shown in Figure 4b. A check for outliers led us to exclude 14 extreme values out of 528 (outlier bounderies were set at ±2 SDs). An ANOVA for repeated measures was carried out on the estimated alignments, with *stimulus*, *distance*, and *replication* as within factors. Both factors *stimuli* (*F*_(1, 21)_ = 71.16, *p* < 0.001,η^2^_p_ = 0.77) and *distance* (*F*_(1, 21)_ = 17.65, *p* < 0.001, η^2^_p_ = 0.46) determined significant effects. None of the interactions reached statistical significance.

Experiment 2 was designed to test whether the Poggendorff illusion is present in Lille's version of the *Descent from the cross*. Results confirm that the illusion is present in such painting as observers’ adjustments made from both distances are statistically different from the geometrical alignment made by the artist: *Lille* distance far (M = −14.61) *t*(21) = −3.69, *p* = .001,*d* = −.78; distance near (M = −35.77) *t*(21) = −5.87 *p* < .001,*d* = −1.25.

Adjustments made for the Poggendorff-like configuration differed from the point of geometrical alignment for the distance far—*t*(21) = 4.49, *p* < .001, *d* = 1.05—but not for the distance near as shown in Figure 4b (*p* = .7). Moreover, the difference in the adjustments for the two types of stimuli suggests that figural complexity of the image plays an important role in the perceptual outcome of the illusion (Spivey-Knowlton & Bridgeman, 1993).

## General Discussion

**Figure 5.**
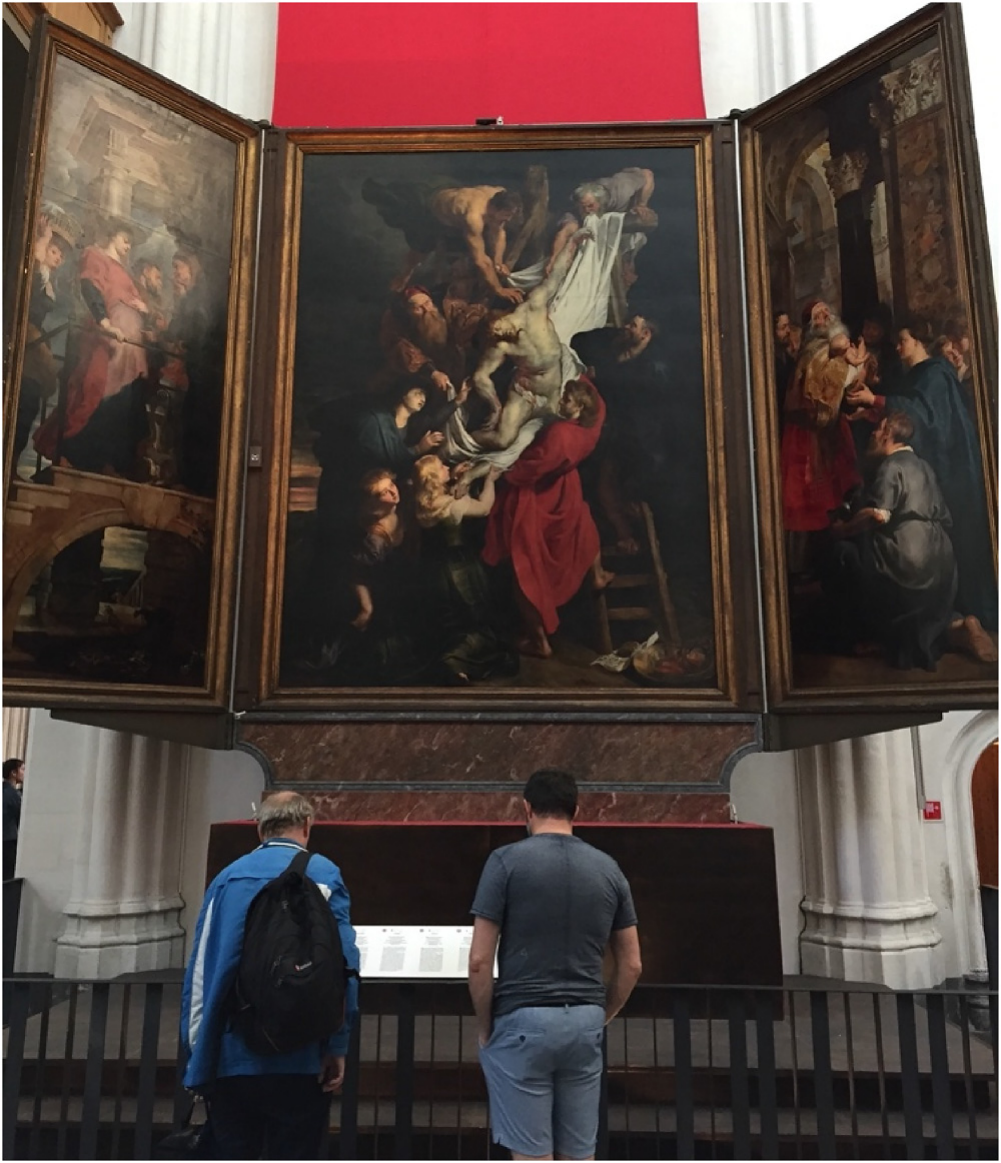
The current location of the altarpiece in Antwerp Cathedral.

The experiments we presented show that the internal configurations of the two *Descents* can generate a Poggendorff illusion. However, only the Antwerp painting shows a geometrical misalignment, and this alleged correction for a Poggendorff illusion is both statistically and perceptually different from the perceptual alignment made by our participants. This fact, combined with the fact that no correction has been made to perceptually align the two visible portions of the ladder's left-side rail in Lille's *Descent from the cross* allows us to draw the conclusion that Rubens did not misalign the two visible portions of the ladder's right rail in the Antwerp masterpiece to correct for the Poggendorff illusion. In fact, by looking at the actual painting in the cathedral (Figure 5), the misalignment chosen by Rubens is clearly visible, yet it is not a detail that disturbs the beauty of this masterpiece. The question, therefore, is why Rubens chose to misalign the upper portion of the ladder's right-side rail. It is most unlikely that Rubens made a mistake. Hence, as we see it, there is only one answer to the aforementioned question, and this is related to the layout of the painting.

Rubens various versions of the *Descent from the Cross* are theatrical machines, in which every character fulfills a role also in terms of weight, balance, and dynamicity (Arnheim, 1974). In the Antwerp masterpiece, there is an ascending movement from bottom left to top right (and of course a descending movement from top right to bottom left); there are also two pivotal characters in the central part of the painting which entertain an action in the opposite direction, and which are most likely to catch the viewer's immediate attention: St. John dressed in red and the pale figure of Christ. Christ is the real core of this amazing theatrical machine, participating in both the left-to-right ascending movement (or right-to-left descending movement) and in the central action, the latter parallel to the ladder on which St. John's foot is positioned. The right rail of the ladder is the troublesome detail on which we are focusing.

**Figure 6.**
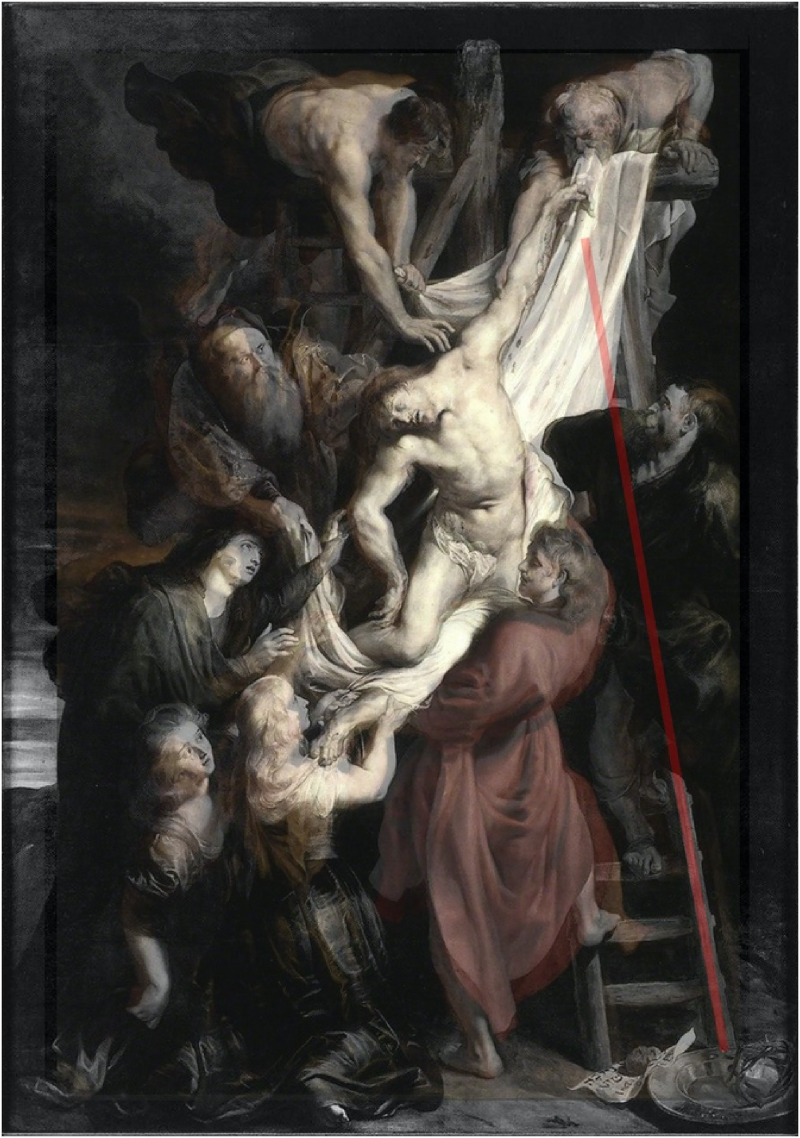
In this image, the Courtauld *Descent* is superimposed on the Antwerp painting. The digital images were scaled such that the two representations of Christ would overlap almost perfectly. The Antwerp painting is rendered in gray scale, while the Courtauld painting is in color but semitransparent. The characters in the Antwerp painting are closer to each other but the shrouds in the two paintings practically overlap. Hence, in the Antwerp painting, the upper part of the ladder on the right would have been completely hidden, hadn't Rubens misaligned the upper portion of its right rail.

Rubens studied in detail the setup of this theatrical machine in the Courtauld oil sketch (Figure 1c), of which the Antwerp masterpiece looks like a rather precise enlargement. In Figure 6, the Courtauld oil sketch is superimposed as a semitransparent layer on top of a grayscale rendering of the Antwerp masterpiece, showing how these would map on a canvas of the same size. As one can see, the Antwerp composition is much “tighter”; the ladder's right rail would have fallen underneath the white cloth. Is the ladder such an important detail that it could not be ignored?

**Figure 7.**
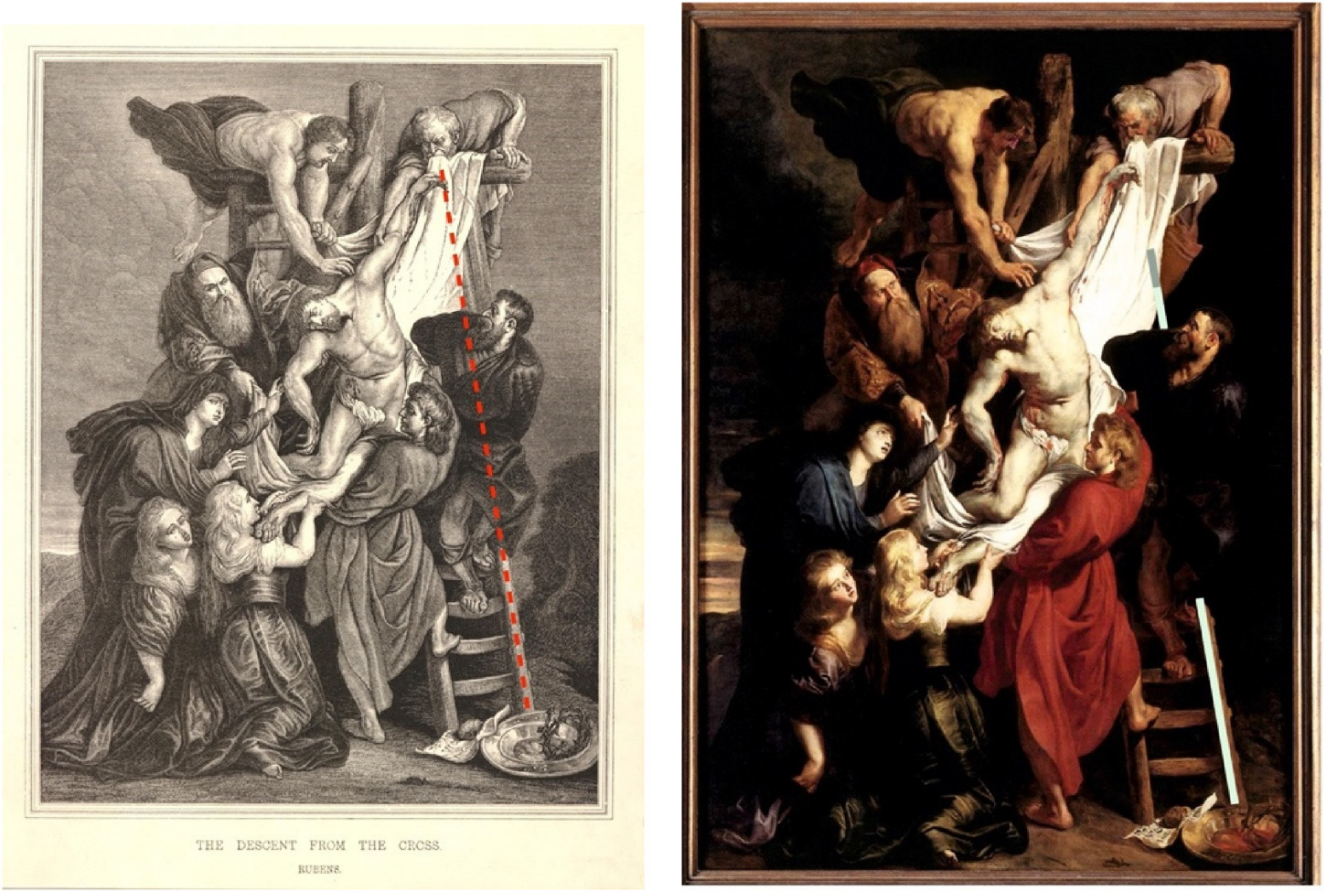
(a) A mid-19th-century wood print copy of the *Descent* from Antwerp, most likely the copy of a copy (see text and footnote; *© The Trustees of the British Museum. Shared under a Creative Commons Attribution-NonCommercial-ShareAlike 4.0 International (CC BY-NC-SA 4.0) licence*). (b) The mean adjustment derived from the experiment is conducted by Zavagno et al. (2015) with book-size digital stimuli.

The answer is both *no* and *yes*. *No*, in the sense that the ladder is not a relevant feature in the evangelical narrative. *Yes*, because it is a crucial component of Ruben’s theatrical machine: geometrical collinearity would have jeopardized the fine dynamic equilibrium of the entire painting, as the ladder in question would appear falling backwards. It was therefore necessary to portray its continuation, given also that the left rail is only visible in its lower part. Figure 7a shows a wood engraving by Édouard Manche dated around the mid-19th century. In this print, the draughtsman displaces the upper portion of the right rail, but only slightly to the right. It is curious that he did not reproduce Ruben’s displacement; but it is even more curious that this displacement corresponds to the mean adjustment found in the experiment conducted by Zavagno et al. (2015), in which the stimuli (renderings of the Antwerp *Descent*) were presented on a computer screen, hence book size compatible. One might be tempted to say that the draughtsman corrected for the Poggendorff illusion as shown in Figure 7a. We are more cautious, and suggest that the misalignment served for another purpose, that is to show the upper part of the right rail, which would have otherwise disappeared under the shroud, as exemplified by the red dotted line. Moreover, the coincidence between the two misalignments shown in in Figure 7(a) and (b) could be just a “happy” one, in the sense that we do not know what Manche actually copied. The real thing or is his a copy of a copy^1^? Let's play with the idea that Manche copied from the original painting in Antwerp: in such a case we suspect that his correction for the Poggendorff illusion would have been more similar to the one shown in Figure 2a, which, however, would render the upper portion of the rail barely visible. Our hypothesis is that Manche misaligned the upper portion of the rail just as much to avoid the impression of a ladder falling backwards.

Getting back to Rubens, one might think that he could have reduced the white cloth. Such cloth, however, is an important detail in the evangelical narrative, as it represents the shroud with which Christ's body was collected and wrapped in. Notice of the existence of the shroud dates to 1353, referring to the relic now conserved in Turin, Italy. Hence, it was not a detail to be overlooked. Moreover, from a compositional point of view, the shroud acts like a slide for the dead body.

Our conclusion is that the evidence against the hypothesis that Rubens adjusted for a Poggendorff illusion is rather strong. Such hypothesis would make sense only if the ladder was a crucial element in the evangelic narrative. Ruben’s misalignment is, instead, more likely related to the dynamics of the theatrical drama he was narrating, in which an uncertain, yet stable equilibrium is staged. Hence, he needed to show the upper portion of the right rail, so that the theatrical machine he invented would not appear to be collapsing.

## Notes

1. Given that the orientation of the printed scene is the same as in the original painting, it could well be that Manche's woodcut was derived by copying another print, which would have been most likely inverted, given that in copying the works of painters, most talented draughtsmen create the matrix by directly copying the painting. Hence, the print derived from the matrix will have the scene inverted by 180° (Zavagno & Massironi, 2006).
